# Comparison of White Blood Cell Scintigraphy, FDG PET/CT and MRI in Suspected Diabetic Foot Infection: Results of a Large Retrospective Multicenter Study

**DOI:** 10.3390/jcm9061645

**Published:** 2020-05-30

**Authors:** Chiara Lauri, Andor W.J.M. Glaudemans, Giuseppe Campagna, Zohar Keidar, Marina Muchnik Kurash, Stamata Georga, Georgios Arsos, Edel Noriega-Álvarez, Giuseppe Argento, Thomas C. Kwee, Riemer H.J.A. Slart, Alberto Signore

**Affiliations:** 1 Nuclear Medicine Unit, Department of Medical-Surgical Sciences and of Translational Medicine, “Sapienza” University of Rome, 00161 Rome, Italy; chialau84@hotmail.it (C.L.); gius.campagna@gmail.com (G.C.); 2 Department of Nuclear Medicine and Molecular Imaging, University of Groningen, University Medical Center Groningen, 9700 Groningen, The Netherlands; a.w.j.m.glaudemans@umcg.nl (A.W.J.M.G.); t.c.kwee@umcg.nl (T.C.K.); r.h.j.a.slart@umcg.nl (R.H.J.A.S.); 3 Department of Nuclear Medicine, Rambam Health Care Campus, 3109601 Haifa, Israel; z_keidar@rambam.health.gov.il (Z.K.); kurash.marina@gmail.com (M.M.K.); 4 3rd Department of Nuclear Medicine, Aristotle University Medical School, Papageorgiou General Hospital, 56403 Thessaloniki, Greece; matageorga@gmail.com (S.G.); garsos@auth.gr (G.A.); 5 Department of Nuclear Medicine, University Hospital of Ciudad Real, 13005 Ciudad Real, Spain; edelnoriega@gmail.com; 6 Radiology Unit, Sant’Andrea University Hospital, 00189 Rome, Italy; giuseppe.argento@uniroma1.it; 7 Department of Biomedical Photonic Imaging, Faculty of Science and Technology, University of Twente, 7500 Enschede, The Netherlands

**Keywords:** diabetic foot, infection, diagnosis, WBC scintigraphy, FDG PET/CT, MRI

## Abstract

Diabetic foot infections (DFIs) represent one of the most frequent and disabling morbidities of longstanding diabetes; therefore, early diagnosis is mandatory. The aim of this multicenter retrospective study was to compare the diagnostic accuracy of white blood cell scintigraphy (WBC), ^18^F-fluorodeoxyglucose positron emission tomography/computed tomography ((^18^F) FDG PET/CT), and Magnetic Resonance Imaging (MRI) in patients with suspected DFI. Images and clinical data from 251 patients enrolled by five centers were collected in order to calculate the sensitivity, specificity, and accuracy of WBC, FDG, and MRI in diagnosing osteomyelitis (OM), soft-tissue infection (STI), and Charcot osteoarthropathy. In OM, WBC acquired following the European Society of Nuclear Medicine (EANM) guidelines was more specific and accurate than MRI (91.9% vs. 70.7%, *p* < 0.0001 and 86.2% vs. 67.1%, *p* = 0.003, respectively). In STI, both FDG and WBC achieved a significantly higher specificity than MRI (97.9% and 95.7% vs. 83.6%, *p* = 0.04 and *p* = 0.018, respectively). In Charcot, both MRI and WBC demonstrated a significantly higher specificity and accuracy than FDG (88.2% and 89.3% vs. 62.5%, *p* = 0.0009; 80.3% and 87.9% vs. 62.1%, *p* < 0.02, respectively). Moreover, in Charcot, WBC was more specific than MRI (89.3% vs. 88.2% *p* < 0.0001). Given the limitations of a retrospective study, WBC using EANM guidelines was shown to be the most reliable imaging modality to differentiate between OM, STI, and Charcot in patients with suspected DFI.

## 1. Introduction

Diabetes-related foot complications represent some of the most frequent and disabling morbidities of longstanding diabetes and are associated with prolonged hospitalization and high social costs [1,2,3,4,5]. Patients with peripheral neuropathy and microvascular impairment have an increased risk of developing an ulcer that could represent a breach for the entry of bacteria, thus potentially causing an infection. The process initially involves the soft tissues (STs) of the foot, and later it could spread in depth for contiguity and reach the bone, leading to diabetic foot osteomyelitis (DFO). Osteomyelitis (OM) is a severe complication for the diabetic patient, with a high risk of amputation and mortality rates [6,7,8]. Considering that more than 50% of wounds are infected at their presentation [2], the prevention of foot ulcers, early diagnosis, and appropriate and prompt treatment are mandatory in order to avoid such a complication and amputation [9,10]. Moreover, about 2.5% of diabetic patients have a Charcot foot, a progressive degenerative disease of the musculoskeletal system characterized by destruction of the bony architecture, which usually involves tarsal and metatarsal joints [11]. The presence of this neuro-ostearthropathy further complicates the diagnostic approach to diabetic foot infection (DFI) since this condition may coexist with the presence of ulcers, thus making the correct diagnosis difficult to achieve. Nevertheless, a differential diagnosis between Charcot, OM, and soft tissue infection (STI) is crucial for the correct management in patients suspected of DFI.

The gold standard examination for the diagnosis of OM is represented by the isolation of the pathogen using microbiological assays. Bone biopsy, however, is an invasive procedure and its reliability strongly depends on the quality of the specimen obtained in the aseptic procedure. Moreover, clinical examination and biochemical inflammatory markers are often non-specific and do not allow a differentiation between infection and inflammation [12,13]. Several diagnostic imaging tests are currently available, including both radiological and nuclear medicine (NM) non-invasive imaging modalities. Despite a consensus document for the diagnosis of peripheral bone infections being recently published, it does not specifically address DFI [14]. Therefore, at present, no clear consensus on the most appropriate imaging technique in suspected DFI exists [7,15,16]. Magnetic resonance imaging (MRI) is the best radiological modality for the assessment of soft tissue abnormalities and may be able to differentiate between STI and OM. However, its specificity can be reduced in the presence of bone marrow edema, synovial effusion, dislocation, bony destruction, and loss of discernible bone and joint margins since they may characterize neuropathic joint disease as well as OM [17]. The NM gold standard in this field is represented by white blood cell scintigraphy (WBC), which provides an in vivo demonstration of the presence of the infective focus. The European Society of Nuclear Medicine (EANM) has previously published guidelines [14,18,19,20,21,22], aiming to define the correct labelling procedure, acquisition, and interpretation criteria for WBC. However, currently, not all institutes follow these recommended standards. The use of ^18^F-fluorodeoxyglucose ((^18^F) FDG) positron emission tomography combined with computed tomography (PET/CT) has gained a role in several indications in the field of infection and inflammation [21]. This technique is widely available and has the advantages of a short acquisition time, high image resolution, and no need of blood handling and manipulation. FDG, however, accumulates in both infection and sterile inflammation since all the involved cells use glucose as a source of energy [21]. Therefore, it is not clear if FDG PET/CT is adequate enough to discriminate among the different foot complications in diabetic patients [15]. In a recently published systematic review and meta-analysis comparing WBC, FDG PET/CT, and MRI in DFO, it emerged that the sensitivity is approximately 90% for all imaging modalities, with ^99m^Technetium-hexamethylpropyleneamine oxime (^99m^Tc-HMPAO)-labelled WBC scintigraphy demonstrating the highest specificity, followed by FDG PET/CT, MRI, and ^111^Indium (^111^In) oxine-labelled WBC [23].

To the best of our knowledge, no multicenter studies are available in the literature comparing these imaging modalities. Therefore, the aim of this retrospective study was to compare the accuracy of WBC scintigraphy, FDG PET/CT, and MRI in differentiating OM, STI, and Charcot in patients with suspected DFI. In particular, our primary end point was to evaluate the diagnostic approaches adopted among different centers in daily practice, aiming to provide a panoramic view on the diagnostic management in different countries. Moreover, since it is well known that the accuracy of WBC scintigraphy relies on the application of the correct acquisitions protocols, image display, and interpretation criteria, the secondary end point of this study was to evaluate, on a multicenter scale, the impact of recently published EANM guidelines [20] on the diagnosis of DFI.

## 2. Experimental Section

### 2.1. Materials and Methods

This retrospective multicenter study included five centers from The Netherlands, Italy, Israel, Greece, and Spain. Data from consecutive patients affected by diabetic foot complications between June 2008 and June 2014 were locally collected by each center, then merged in a single central database, and processed using SPSS statistic software. This study was approved by each local ethical committee and of the coordinating center (Groningen, the Netherlands).

#### 2.1.1. Patients

Patients were retrospectively recruited by radiology and NM departments of each center where they were sent by the respective local diabetic foot units (DFUs), between 2008 and 2014, for the study of suspected DFI. The following inclusion and exclusion criteria were adopted in this study:

Inclusion criteria (the first four items were mandatory):

- Type 1 or type 2 diabetes treated with oral medications or insulin;
- Suspected DFI based on clinical presentation of foot wounds according to Perfusion, Extent, Depth, Infection, Sensation of Infectious Diseases Society of America (PEDIS/IDSA) classification [1];
- At least one out of the three imaging modalities performed for suspected DFI;
- Final diagnosis provided by gold standard;
- Bony abnormalities detected by plain radiographs;
- Palpable bone at “probe-to-bone test” (in presence of open wounds);
- Raised inflammatory markers.

Exclusion criteria:

- Lack of information on final diagnosis;
- Patients lost at clinical follow-up.

Demographic and laboratory data, including gender, age, type of diabetes, medical history, biochemistry, microbiology, histopathology, treatments, and final diagnosis, were collected by each center. In patients in whom more than one infection was investigated, each episode was considered as an individual event with corresponding imaging.

Patients were clinically or surgically managed by the DFUs of each center following their own protocols.

#### 2.1.2. Imaging Modalities and Analysis

Information regarding the camera type, manufacturer, year of manufactory, the use of markers or contrast, details of the compounds, and sequences for MRI were recorded. For NM examinations, information on radiopharmaceutical, administered activity, type of gamma-camera, equipped or not with single-photon emission tomography/computed tomography (SPECT/CT), PET/CT camera systems, and exact protocols of acquisition were also specified by each center. All acquisition details and DICOM files had to be available in order to include the patients in the study. NM images were examined by two experienced NM physicians. Discordant cases were resolved by consensus. All MRI scans were evaluated by an experienced radiologist. All readers were blinded for clinical details. The following scoring method was used for each diagnostic tool in order to classify the outcome: 0 = negative/sterile inflammation; 1 = OM; 2 = STI; 3 = Charcot. If two or more conditions were concomitant in the same patient, the most clinically relevant was considered (e.g., OM + Charcot = OM; OM + STI = OM; STI + Charcot = STI).

For each scan, the location of disease (in forefoot or mid/hindfoot) was recorded.

#### 2.1.3. Interpretation Criteria for WBC Scintigraphy

- Acquired according to EANM recommendations: Interpretation criteria were used as recommended by the guidelines [20] and obtained by both visual and, in equivocal cases, semi-quantitative analysis by drawing region of interests (ROIs) on target (T) and contralateral side as background (B) in order to calculate the T/B ratio. OM was defined when WBC focal accumulation at 20–24 h was higher in intensity than at 3–4 h. STI was defined when WBC focal or diffuse accumulation at 20–24 h was lower than at 3–4 h. Charcot was defined when diffuse WBC accumulation at 20–24 h was similar or decreased compared to the uptake at 3–4 h.
- Acquired not according to EANM recommendation: In the case scans were performed with only one time point acquisition, they were classified as follows: OM was defined when the WBC focal accumulation was higher than surrounding tissues. STI was defined when WBC accumulation (both focal or diffuse) was observed in the superficial regions of the foot. Charcot was defined when diffuse WBC accumulation at the mid/hindfoot was observed.

#### 2.1.4. Interpretation Criteria for FDG PET/CT

FDG PET/CT assessment was performed using a visual analysis describing the target areas in terms of intensity (grade 0: no uptake; grade 1: uptake at foot location = contralateral side; grade 2: uptake at foot location > contralateral side), pattern of uptake (focal vs. diffuse), number of foci, and the exact localization of the increased uptake as provided by the CT. We also performed a semi-quantitative analysis using the maximum and mean standardized uptake values (SUVmax, SUVmean) of each area with increased uptake. The SUV_max_ ratio (SUV_max_ of target/SUV_max_ contralateral background) was also calculated. OM was defined when focal or diffuse FDG uptake higher than the contralateral side was visible on the bone structure with or without soft tissue involvement. STI was defined when focal or diffuse FDG uptake (grade 1 or 2) was visible only in the soft tissues without bony involvement. Charcot was defined when diffuse FDG uptake (grade 1 or 2) was visible involving tarsal and metatarsal joints and associated with bone destruction on CT.

#### 2.1.5. Interpretation Criteria for MRI

MRI was evaluated for primary and secondary signs of OM, according to the literature [24,25,26]. Briefly, OM was defined in the presence of low medullary bone marrow signal in a geographic confluent pattern on T1-weighted imaging (T1w), concordant with abnormal (high) signal at fat-suppressed T2-weighted (T2w) and post-gadolinium (Gd) T1w imaging.

Secondary signs of OM were also evaluated [24]:

(a) Ulcer (skin interruption with raised margins and associated soft tissue defect);
(b) Sinus tract (“tram track” pattern post-Gd enhancement);
(c) Cellulitis (skin thickening and soft tissue edema with low T1 and T2 signal and post-Gd enhancement);
(d) Abscesses (signal intensity of fluid with post-Gd peripheral rim-like enhancement);
(e) Gangrene (post-Gd non-enhancing area of devitalized tissue that is sharply demarcated from surrounding viable tissue, without (dry gangrene) or with air bubbles in the soft tissue (wet gangrene));
(f) Tenosynovitis (area of post-Gd peri-tendinous enhancement coursing through an area of cellulitis and adjacent to an infected ulcer).

STI was considered present if at least one of the previously described findings was observed without any radiological signs of bone involvement. Charcot was defined as the presence of soft tissue edema, fluid collections, effusions, bone marrow abnormalities, post-Gd peri-articular soft-tissue, and bone marrow enhancement (typically in the tarsal-metatarsal and metatarsal-phalangeal joints), associated with deformities and osseous fragmentation.

#### 2.1.6. Therapeutic Management

The different therapeutic strategies adopted by the DFUs of each local center were recorded when available, and were classified as follows:

- No treatment (including offloading);
- Conventional wound care and topic antibiotic treatment;
- Systemic antibiotic treatment;
- Surgery (debridement or amputation).

When possible, the results of the three imaging modalities were correlated with the treatment received by the patient.

#### 2.1.7. Gold Standard

Bone biopsies were used as gold standard for the final diagnosis of OM and Charcot, independently by the availability of the isolated pathogen. Skin cultures were used for the diagnosis of STI. When bone biopsies or skin cultures were not available, a clinical follow-up of at least 12 months was used in order to confirm or rule out the diagnosis achieved with imaging modalities.

#### 2.1.8. Statistical Analysis

All statistical analyses were performed using SAS version 9.4 and JMP version 14 (SAS Institute, Cary, NC, USA). Categorical variables are expressed as absolute frequencies and percentages. Continuous variables (SUV_max_ and SUV_max_ ratio) are presented in mean ± standard deviation (SD) mean differences across groups (OM, STI, and Charcot) and were compared by generalized linear models. The normality of the residuals was verified by Shapiro–Wilk test and homoscedasticity by the Levene and Brown–Forsythe test. Differences between the final diagnosis (no pathology, OM, and STI) vs. C-reactive protein (CRP) and erythrocytes sedimentation rate (ESR) were evaluated by the Kruskal–Wallis test and Steel–Dwass test using post hoc analysis. The sensitivity, specificity, and diagnostic accuracy of all the three imaging modalities in diagnosing OM, STI, and Charcot foot against the reference standard histology and/or clinical follow-up were performed by routine use of SAS. Comparisons of the sensitivity, specificity, and diagnostic accuracy between the three imaging modalities were performed using the Z test for the equality of two proportions. The Benjamini–Hochberg procedure was applied to check multiple comparisons. The results are reported in a percentage with 95% confidence intervals (CIs). A *p*-value <0.05 was considered statistically significant.

## 3. Results

**Scheme 1:**
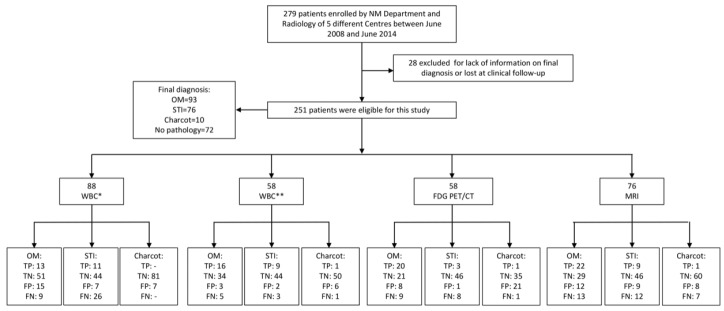
Flow chart on patient selection and imaging modalities. NM: Nuclear Medicine; OM: osteomyelitis; STI: soft tissue infection; WBC: white blood cells scintigraphy; * WBC scans acquired without following European Association of Nuclear Medicine (EANM) guidelines; ** WBC scans acquired following EANM guidelines; ^18^F-fluorodeoxyglucose positron emission tomography/computed tomography (FDG PET/CT); Magnetic Resonance Imaging (MRI); TP: true positives; TN: true negatives; FP: false positives; FN: false negatives; - no patients with a final diagnosis of Charcot foot were studied with * WBC scan.

A total of 251 patients were enrolled in the contributing five centers (Scheme 1). A descriptive analysis of our population is summarized in Table 1.

**Figure 1:**
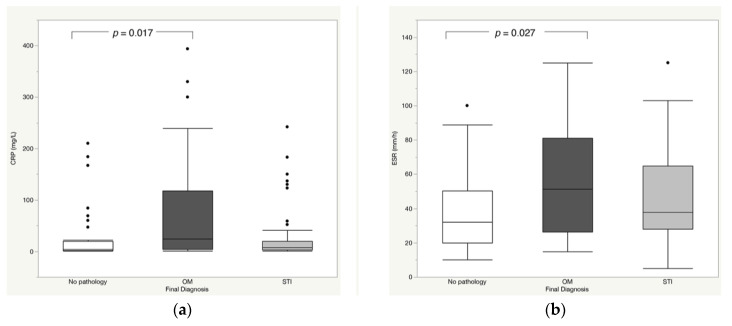
Box plots of C-reactive protein (CRP) (**a**) and erythrocyte sedimentation rate (ESR) (**b**) showing significantly higher values of CRP (median value 24.0 mg/L; min to max: 1–393.80) and ESR (median value 51.5 mm/h; min to max: 15.0–125) compared with non-infected patients (median values of CRP: 4.5 mg/L; min to max: 1–210); median values of ESR: 32.0; min to max: 10–100). OM: osteomyelitis; STI: soft tissue infection.

Post hoc analysis on median values of CRP and ESR showed that they were both significantly higher in patients with OM compared with patients without any infection (*p* = 0.017 and *p* = 0.027 respectively) as illustrated in Figure 1. No similar significant difference was observed between patients with STI and normal subjects and between patients with OM and STI.

Causative pathogens were recorded in 67 patients that underwent skin cultures, and in 14 out of 50 patients who performed (pre- or intra-operative) biopsy; however, biopsy was used as a gold standard for final diagnosis in the other 36 patients in which we could not obtain information on the pathogen causing the infection. In the remaining 121 patients, final diagnosis was assessed with clinical follow-up (see Table 2). OM was found in 93 patients, STI in 76, and Charcot in 10 patients. The remaining 72 subjects had no pathology according the reference standard. Regarding the imaging modalities, 119 patients underwent a WBC scintigraphy, 46 FDG PET/CT, and 59 patients underwent MRI. In 10 patients, both WBC and FDG PET/CT were performed; in 15 patients, both WBC scintigraphy and MRI; and in 2 patients, all three imaging techniques were performed. The diagnostic performances of the three imaging modalities are summarized in Table 3.

### 3.1. WBC Scintigraphy

The mean administered activity of ^99m^Tc-radiolabelled leukocytes was 569.8 ± 116.5 MBq (15.4 ± 3.15 mCi).

**Figure 2:**
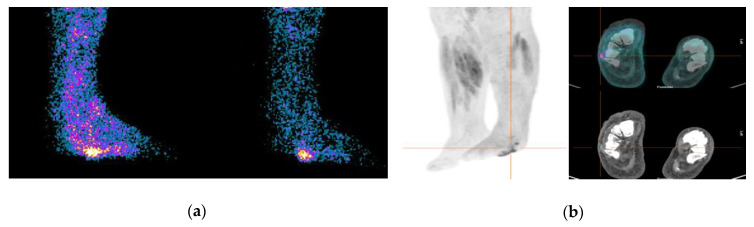
(**a**) Planar images of the WBC scan acquired with correction for ^99m^Technetium (^99m^Tc) decay at 3 h (on the left) and 20 h (on the right). The focal uptake of labelled leukocytes detectable on the plantar surface of the left foot decreases between the 3 and 20 h images, being consistent with the diagnosis of STI; (**b**) Multi Intensity Projection (MIP) and transaxial views of FDG PET/CT, of the same patient, show the focal uptake in the plantar surface of the left foot in correspondence with a cutaneous/subcutaneous ulcer of the soft tissues. Bone and soft tissue biopsies confirmed the diagnosis of STI and the patient was treated with topic antibiotic therapy. WBC: white blood cells scintigraphy; FDG PET/CT, fluorodeoxyglucose positron emission tomography/computed tomography.

Since a significant discrepancy was observed among the different centers regarding the acquisition procedure, we compared the results obtained by centers that applied EANM guidelines with those who did not follow these recommendations. Fifty-eight scans were acquired according to the EANM guidelines for WBC scintigraphy with acquisition times corrected for Tc-99m decay at two (4 and 24 h) or three times points (30’, 3 h, and 20 h) [20] (Figure 2). Eighty-eight scans did not follow these recommendations; in particular, 73 scans (83.0%) were acquired only 4 h post injection (p.i.) and 15 (17.0%) were acquired at 2 time points, 1 and 4 h p.i. Furthermore, given the low number of patients who performed combined SPECT/CT in these two groups that did not allow us to make a comparative analysis, we considered only planar images for the diagnosis, thus exploring the importance of multiple time-point acquisitions.

The sensitivity, specificity, and accuracy of the labelled WBC planar images of the two groups are reported in Table 3.

### 3.2. FDG PET/CT.

FDG PET/CT scans were generally acquired 60 min after the injection of approximately 3 MBq (0.08 mCi)/kg of FDG according to EANM guidelines [21]. Both qualitative assessment (intensity and pattern of uptake) and semiquantitative analysis of FDG uptake were performed (Table 4).

In total, 96.5% of patients with a final diagnosis of OM (29 patients) showed “grade 2 uptake” that was focal in 62.1% (18 patients) of cases and diffuse in the remaining 37.9% (11 patients). The mean values of the SUV_max_ and SUV_max_ ratio were 5.3 ± 2.1 and 3.9 ± 2.4, respectively. All patients with STI (11) showed “grade 2 uptake” and it was focal in 54.5% of cases (6 patients) and diffuse in the remaining 45.5% of cases (5 patients). The mean values of the SUV_max_ and SUV_max_ ratio were respectively 5.4 ± 1.8 and 4.2 ± 2.1. Only two patients with Charcot were studied with FDG PET/CT and they all patients showed “grade 2” and diffuse uptake. The mean values of the SUV_max_ and SUV_max_ ratio were respectively 4.9 ± 1.9 and 4.9 ± 1.6. The semiquantitative analysis did not provide a significant difference in terms of both the SUV_max_ and SUV_max_ ratio between the three groups of patients (*p* = 0.48 and *p* = 0.83, respectively). The sensitivity, specificity, and accuracy for FDG PET/CT are reported in Table 3.

### 3.3. MRI

All centers used similar protocols of acquisitions that included at least T1w, fat-suppressed T2w, and post-Gd T1w sequences, with fat suppression or with subtraction of pre- and post-Gd T1w. Sequences were acquired in at least two perpendicular planes.

The sensitivity, specificity, and accuracy for MRI are reported in Table 3.

### 3.4. Comparison between WBC Scintigraphy, FDG PET/CT, and MRI in Suspected DFI

WBC scintigraphy, in particular if acquired according to EANM guidelines, showed significantly higher specificity and accuracy than MRI (*p* < 0.0001 and *p* = 0.003, respectively) in detecting OM. Moreover, the sensitivity, specificity, and accuracy of WBC scintigraphy were higher than FDG PET/CT, although not statistically significant. In STI, both FDG PET/CT and WBC scintigraphy achieved a significantly higher specificity than MRI (*p* = 0.04 and *p* = 0.018, respectively). The sensitivity of the three imaging modalities in detecting Charcot could not be calculated because of the low number of patients, but both MRI and WBC scintigraphy showed significantly higher specificity and accuracy than FDG PET/CT (*p* = 0.0009 and *p* = 0.029, respectively, for MRI and *p* = 0.0009 and *p* = 0.003, respectively, for the radiolabeled WBC scan). Moreover, WBC scintigraphy provided significantly higher specificity than MRI (*p* < 0.0001) in this condition. However, these results were based on only a small sample size.

### 3.5. Comparison between WBC Scintigraphy Performed according and not according to EANM Guidelines

In both OM and STI, using standardized protocols resulted in an overall increase of the sensitivity (from 59.1 to 76.2% and from 29.7% to 75%, respectively), specificity (from 77.3% to 91.9% and from 86.3% to 95.7%, respectively), and diagnostic accuracy (from 72.7% to 86.2% and from 62.5% to 91.4%, respectively) in comparison to those who did not use these protocols. Statistical significance was reached when comparing the sensitivity (*p* = 0.006) and diagnostic accuracy (*p* < 0.0001) in the evaluation of STI. In Charcot, due to the low number of the subjects (only two patients in the “EANM-approved protocols” group and none in the other), this comparison could not be done.

### 3.6. Comparison between WBC Scintigraphy, FDG PET/CT, and MRI according to the Location of Disease

We compared the performance of the three imaging modalities in the forefoot and mid/hindfoot disorders (Table 5).

Patients without any histopathology results (28.6%) according to the reference and patients with Charcot (only 3.9% of whole population) were excluded. FDG PET/CT was more specific than MRI in detecting STI in mid/hindfoot (*p* = 0.03). For the detection of OM, WBC scintigraphy showed significantly higher sensitivity in the forefoot rather than the mid/hindfoot (*p* = 0.013). Similarly, FDG showed significantly higher sensitivity and accuracy in detecting OM of the forefoot compared to the mid/hindfoot (*p* = 0.026 and *p* = 0.015, respectively).

### 3.7. Correlation between the Findings of Diagnostic Tests and Therapeutic Management

A descriptive analysis of the different therapeutic options conducted in the studied population, according to the final diagnosis, is summarized in Table 6 and Table 7. Most patients with final diagnosis of OM (64.4%) underwent surgery and 32.8% were treated with antibiotics. WBC scintigraphy correctly identified 77% and 83% of these patients, FDG identified 83% and 50% of them, and MRI identified 65% and 73% of them.

By contrast, most patients with a final diagnosis of STI (68.3%) were treated with antibiotics and only 15% underwent surgery. WBC scintigraphy correctly identified 39% and 72% of these patients while MRI identified 47% and 66% of them.

Since we did not observe any statistically significant difference between the three imaging modalities in therapy decision-making, we can conclude that they have similar accuracy in guiding clinicians for the correct management of patients affected by OM or STI.

## 4. Discussion

This retrospective multicenter study is, to the best of our knowledge, the first to compare the diagnostic value of WBC scintigraphy, FDG PET/CT, and MRI in a large population of patients with suspected DFI, with a particular emphasis on the ability of these imaging modalities to differentiate between OM, STI, and Charcot. At present, no similar studies are available in the literature, probably reflecting the heterogeneity in the diagnostic approaches used in patients from different countries. We believe that a consensus on the most appropriate imaging tool for the assessment of OM, STI, and Charcot is necessary in order to standardize the management of these patients in all centers.

Our analysis shows that the radiolabeled WBC scintigraphy, especially if acquired according to EANM guidelines, has an overall high diagnostic performance in OM, STI, and Charcot, in particular, in terms of the specificity and diagnostic accuracy.

It is well known that when adhering to standardized protocols for labelling, acquisition, image display, and interpretation, the accuracy of WBC scintigraphy in detecting different kinds of infection can be increased [20,27,28]. In our population, 58 patients were studied according to the EANM guidelines for WBC image acquisition and display, while 88 scans did not follow these recommendations. In these 88 patients, clear superiority of the WBC scan over FDG PET/CT and MRI did not emerge neither in OM nor STI and Charcot. On the other hand, comparing the results of WBC scintigraphy acquired according to EANM guidelines with FDG PET/CT and MRI, we found that the WBC scan was more accurate and specific than MRI in OM, more specific than MRI in STI, and more specific and accurate than FDG in Charcot. These results emphasize the importance of using a standardized image acquisition protocol as suggested by EANM guidelines [20].

Furthermore, comparing the results of WBC scintigraphies acquired according to the EANM guidelines versus WBC acquired without following these recommendations, we found a statistically significant difference in the sensitivity (*p* = 0.006) and diagnostic accuracy (*p* < 0.0001) in the evaluation of STI. This is extremely important considering that STI in diabetics is a particular type of infection that cannot be managed as a common STI in other areas or in non-diabetic patients [29]. The presence of microangiopathy and neuropathy can impair tissue healing and treatment efficacy, with possible progression in OM. Therefore, the diagnosis of STI must be prompt and accurate. In OM, we also found a better specificity (*p* = 0.06) and accuracy (*p* = 0.05) in patients acquired with the EANM protocol than in patients acquired with non-EANM protocols, despite the statistical significance being low. ^111^In-oxine could also be used as an alternative to ^99m^Tc-HMPAO for the labelling of WBC and it is also able to provide a high accuracy in detecting an infection [19,20]; however, due to its physical characteristics and radiation burden, ^99m^Tc remains the preferred agent.

FDG PET/CT has gained an important role in the diagnosis and follow-up of several infective and inflammatory diseases, but it is still unclear whether it could represent a valid alternative to WBC scintigraphy in DFI [15,21]. Moreover, the diagnostic performance of this modality mostly relies on the CT scan, which allows correct anatomical localization and evaluation of the extent of FDG uptake [30]. In our population, FDG PET/CT showed a higher specificity compared with MRI (respectively 95.7% vs. 83.6%) in detecting STI, especially in the mid/hindfoot. This may be explained by the use of CT, which improves the localization of the uptake to soft tissues, thus facilitating the achievement of a correct diagnosis and justifying the low number of false positive results with FDG PET/CT. Nevertheless, as explained above, in STI, the sensitivity is more important than the specificity and, to this regard, WBC showed better sensitivity than FDG. Moreover, we found a good sensitivity, specificity, and accuracy of FDG PET/CT in detecting pedal OM, especially in the forefoot. Although precise interpretative criteria exist for WBC scintigraphy, they are not applicable for FDG PET/CT. Several authors investigated the possible role of SUV measurements in the diagnosis of infectious processes, but the results are discordant among different studies [31,32,33]. For example, Basu et al. [31] found higher SUV_max_ values in patients with OM compared with patients with Charcot and uncomplicated DF (2.9–6.2 vs. 0.7–2.4 vs. 0.2–0.7), concluding that SUV could be a useful parameter for differentiating these conditions. Conversely, other authors did not find any significant correlation between SUV_max_ values and STI, OM, or Charcot, concluding that SUV alone is not sufficient for diagnosis [15,32,34]. In agreement with this view, in our population, SUV_max_ and SUV_max_ ratios did not significantly differ among patients with OM, STI, and Charcot. Qualitatively, we found a diffuse tracer uptake in all patients with Charcot and a focal uptake in the majority of patients affected by OM. In STI, we found more or less the same proportion of patients with focal and diffuse uptake, thus a precise pattern cannot be determined in this specific condition. Additionally, the evaluation of intensity was found to not be useful in discriminating between these three conditions since “grade 2 uptake” was present in almost all patients studied regardless of the final diagnosis.

In accordance with published meta-analysis and systematic reviews [23,34,35], we found that MRI was not superior to NM imaging modalities, showing high false positive and false negative rates. In the literature, the reported sensitivity of MRI in defining OM ranges between 77.0% and 100.0% [36,37,38], and, despite its the excellent spatial resolution and natural contrast between different structures, there are several conditions in which MRI is not accurate (e.g., in post-traumatic or post-operative phases or in the presence of lower limb ischemia). In this study, we did not exclude patients with peripheral ischemia, although this condition might have influenced the outcome of MRI [39] and, therefore, this aspect should be considered when comparing the diagnostic performance of the three different modalities. However, lower limb ischemia is a common diagnostic problem that also affects the correct interpretation of WBC scintigraphy, especially for the detection of STI, since a reduced vascular supply could impair leukocytes’ recruitment into infected sites [29]. Moreover, in the diabetic foot, the possible presence of Charcot and mechanical stress can be responsible for changes in the bone marrow or soft tissue intensity that could be erroneously interpreted as an OM, thus impairing the specificity of MRI [40,41,42].

Although it was not a specific aim of our study, we also analyzed the relationship between inflammatory markers and the final diagnosis of infection and found significantly higher values of CRP and ESR in patients with OM compared to patients without any infection. However, we did not find a similar significant difference between patients with STI and normal subjects and between patients with OM and STI. Moreover, aiming to assess whether these values could be predictive of imaging outcomes, the analysis revealed that they are not reliable tools to predict a positive result of MRI, WBC scintigraphy, and FDG PET/CT. Indeed, the role of inflammatory markers is much debated and, although they are usually elevated in infections, they are not able to discriminate the kind of infection and to evaluate its severity, which could be accurately and non-invasively assessed only by advanced imaging.

Despite the large number of patients recruited, this study has several limitations. First, the retrospective nature resulted in a wide heterogeneity of patient selection and diagnostic and therapeutic approaches adopted among the different centers. Often, the choice of one imaging modality rather than another was based on local center availability or waiting lists for the scan, and this bias could of course reflect the comparability of different imaging modalities. Moreover, the different acquisition protocols adopted of both NM and radiological images could negatively affect the results, as shown in the group of patients who performed the WBC scan without following the EANM guideline. Another important limitation is the lack of completeness of the data. In several patients, we missed clinical or histopathological data. In addition, the lack of SPECT/CT in the majority of cases did not allow us to consider this imaging modality in the analysis, but only planar images. Finally, the low number of patients with Charcot does not allow final conclusions to be drawn regarding the best diagnostic approach in this condition. Moreover, it is well known that bone marrow scintigraphy has a central role in handling doubtful WBC scintigraphy, and this modality is particularly important in the evaluation of mid-hindfoot disorders in order to differentiate between an infection from a bone marrow expansion, which is typical of Charcot foot. Unfortunately, given the retrospective nature of this study, only a few patients performed bone marrow scintigraphy in addition to WBC scintigraphy, thus not allowing an evaluation of the added value of this combined approach on the diagnosis.

Nevertheless, retrospective multicenter studies are of clinical value because they consist of data from centers with different experiences, protocols, and equipment, thus representing the actual real situation in daily clinical practice for the diagnosis of DFI. In this context, our study underlines the need for the standardization of acquisition protocols and of interpretation criteria, aiming to correctly manage patients with suspected DFI. Professionals requesting these imaging tests should be specialists in the management of DF to reduce the diagnostic and therapeutic variability of IDFs. 

The recent availability of PET/MRI may gain an important role in defining the different complications of the diabetic foot in the near future and it will hopefully solve these challenging clinical scenarios [43,44,45].

## 5. Conclusions

This retrospective study confirms the superiority of WBC scintigraphy over other imaging modalities in discriminating pedal OM, STI, and Charcot. In particular, when EANM guidelines are applied, this examination results in high sensitivity, specificity, and accuracy. Additional randomized powered prospective studies comparing these three imaging modalities, and possibly SPECT/CT and PET/MRI in the same patient, are needed to provide a basis for a proper evidence-based multi-modality diagnostic algorithm.

## Figures and Tables

**Table 1:** General characteristics of the study population.

	Age (Years) (Mean ± SD)	Gender (F/M) (%)	DM1/DM2 (%)	Glycaemia (mmol/L) (Mean ± SD)	Hb1Ac (%) (Mean ± SD)	ESR (mm/h) (Mean ± SD)	CRP (mg/L) (Mean ± SD)
All patients (251)	60.7 ± 10.8	34.3/65.7	17.1/82.9	9.1 ± 3.4	8.0 ± 1.9	47.3 ± 28.5	43.0 ± 73.1
WBC * (88)	64.2 ± 10.9	44.3/55.7	15.9/84.1	8.5 ± 2.8	7.7 ± 1.6	45.6 ± 28.0	14.3 ± 32.8
WBC ** (58)	57.2 ± 10.3	32.7/67.3	15.5/84.5	9.7 ± 4.2	9.2 ± 2.6	56.6 ± 35.9	73.4 ± 58.6
MRI (76)	59.2 ± 10.6	25.0/75.0	22.4/77.6	10.5 ± 4.0	8.2 ± 2. 2	48.2 ± 27.5	87.4 ± 100
FDG (58)	58.0 ± 9.0	29.3/70.7	10.3/89.7	7.8 ± 2.6	8.3 ± 1.9	n.r.	n.r.
OM (93)	59.6 ± 11.1	26.9/73.1	20.4/79.6	9.5 ± 3.3	8.4 ± 1.9	55.9 ± 32.1	68.3 ± 93.7
STI (76)	62.3 ± 10.01	38.1/61.9	19.7/80.3	9.0 ± 3.6	7.8 ± 1.7	46.7 ± 26.9	27.9 ± 53.4
Charcot (10)	56.5 ± 10.1	20.0/80.0	20.0/80.0	n.r.	7.4 ± 1.7	n.r.	42.8 ± 71.5
No pathology (72)	60.7 ± 10.9	43.0/57.0	9.7/90.3	8.5 ± 3.2	7.7 ± 1.9	37.6 ± 23.1	27.7 ± 53.7

In brackets: number of patients; SD: Standard Deviation; F: females; M: males; DM1: Type 1 Diabetes Mellitus; DM2: Type 2 Diabetes Mellitus; Hb1Ac: glycated hemoglobin; ESR: Erythrocytes Sedimentation Rate; CRP: C Reactive Protein; * WBC: scans acquired without following EANM guidelines; ** WBC: scans acquired following EANM guidelines; n.r: not reliable because data available in less than 10 patients. MRI, magnetic resonance imaging; FDG, fluorodeoxyglucose; OM: osteomyelitis; STI: soft tissue infection; WBC: white blood cells scintigraphy; EANM, European Society of Nuclear Medicine.

**Table 2:** Microbiology and histopathological findings.

Cultures from Aspirates (in 67 Patients)	Cultures from Bone Biopsies (in 14 Patients)
Negative: 13.4%	Negative: 28.6% *Polymicrobial*: 28.6% *Staph. aureus*: 28.6% *Pseud. aeruginosa*: 14.2%
*Staph. aureus*: 26.9%	
*Polymicrobial*: 23.8%	
*Strept. epidermidis*: 10.4%	
*P. aeruginosa*: 7.5%	
*Acinetobacter*: 4.5%	
*Staph. haemolyticus*: 4.5%	
*E. faecalis*: 3.0%	
*Proteus mirabilis*: 1.5%	
*E. coli*: 1.5%	
*Strept. agalactiae*: 1.5%	
*Citrobacter freundii*: 1.5%	

**Table 3:** Overview of the performance of diagnostic imaging tests in detecting OM, STI, and Charcot.

	Sensitivity (%) (95% CI)	Specificity (%) (95% CI)	Accuracy (%) (95% CI)
OM (WBC *)	59.1 (38.5–79.6)	77.3 (67.2–87.4)	72.7 (63.4–82.0)
STI (WBC *)	29.7 (15.0–44.5)	86.3 (76.8–95.7)	62.5 (52.4–72.6)
Charcot (WBC *)	n.c.	92.0 (86.4–97.7)	n.c.
OM (WBC-EANM **)	76.2 (58.0–94.4)	91.9 (83.1–100)	86.2 (77.3–95.1)
STI (WBC-EANM **)	75.0 (50.5–99.5)	95.7 (88.9–100)	91.4 (84.2–98.6)
Charcot (WBC-EANM **)	n.c.	89.3 (81.2–97.4)	87.9 (79.5–96.3)
OM (FDG PET/CT)	69.0 (52.1–85.8)	72.4 (56.1–88.7)	70.7 (59.0–82.4)
STI (FDG PET/CT)	27.3 (1.0–54.0)	97.9 (93.7–100)	84.5 (75.2–93.8)
Charcot (FDG PET/CT)	n.c.	62.5 (49.8–75.2)	62.1 (49.6–74.6)
OM (MRI)	62.9 (46.8–78.9)	70.7 (56.8–84.7)	67.1 (56.5–77.7)
STI (MRI)	42.9 (21.7–64.0)	83.6 (73.9–93.4)	72.4(62.3–82.4)
Charcot (MRI)	n.c.	88.2 (80.6–95.9)	80.3 (71.3–89.2)

* WBC scans acquired without following EANM guidelines; ** WBC scans acquired following EANM guidelines; n.c. = not calculated because of the low number of patients. FDG PET/CT, fluorodeoxyglucose positron emission tomography/computed tomography.

**Table 4:** Pattern of FDG PET/CT in the study population.

Final Diagnosis	Intensity		SUV_max_ (Mean ± SD ^1^)	SUV_max_ Ratio (Mean ± SD ^1^)
	Focal Uptake (%)	Diffuse Uptake (%)		
OM	62.1	37.9	5.3 ± 2.1	3.9 ± 2.4
STI	54.5	45.5	5.4 ± 1.8	4.2 ± 2.1
Charcot	0	100	4.9 ± 1.9	4.9 ± 1.6

**^1^** SD: Standard deviation.

**Table 5:** Comparison between the performance of WBC scintigraphy, FDG PET/CT, and MRI in detecting OM and STI according to the location.

	Sensitivity (%) (95% CI)		Specificity (%) (95% CI)		Accuracy (%) (95% CI)	
	Forefoot	Mid-Hindfoot	Forefoot	Mid-Hindfoot	Forefoot	Mid-Hindfoot
OM (WBC-EANM **)	78.6 (57.1–100)	71.4 (38.0–100)	88.9 (68.4–100)	85.7 (59.8–100)	82.6 (67.1–98.1)	78.6 (57.1–100)
STI (WBC-EANM **)	85.7 (59.8–100)	60.0 (17.1–100)	87.5 (71.3–100)	100 (100–100)	87.0 (73.2–100)	85.7 (67.4–100)
OM (FDG PET/CT)	91.7 (76.0–100)	52.9 (29.2–76.7)	60.0 (17.1–100)	72.7 (46.4–99.0)	82.4 (64.2–100)	60.7 (42.6–78.8)
STI (FDG PET/CT)	50.0 (1.0–99.0)	14.3 (0.0–40.2)	100 (100–100)	100 (100–100)	88.2 (75.9–100)	78.6 (63.4–93.8)
OM (MRI)	72.0 (54.0–89.6)	40.0 (9.6–70.4)	61.9 (41.1–82.7)	76.9 (54.0–99.8)	67.4 (53.8–80.9)	60.9 (40.9–80.8)
STI (MRI)	42.9 (16.9–68.8)	42.9 (6.2–79.5)	93.8 (85.4–100)	75.0 (53.8–96.2)	78.3 (66.3–90.2)	65.2 (45.7–84.7)

** WBC scans acquired following EANM guidelines.

**Table 6:** Different therapeutic strategies in patients with final imaging diagnosis of OM.

Therapeutic Strategy in OM	Diagnosis according to Imaging Modalities (%)			
Conventional wound care and topic antibiotic treatment 2.8%	WBC			
	Negative 0%	OM 100%	STI 0%	Charcot 0%
	FDG (no patients)			
	MRI			
	Negative 0%	OM 0%	STI 0%	Charcot 100%
Systemic antibiotic treatment 32.8%	WBC			
	Negative 0%	OM 83%	STI 0%	Charcot 17%
	FDG			
	Negative 0%	OM 50%	STI 0%	Charcot 50%
	MRI			
	Negative 9%	OM 73%	STI 9%	Charcot 9%
Surgery (debridement or amputation) 64.4%	WBC			
	Negative 3%	OM 77%	STI 10%	Charcot 10%
	FDG			
	Negative 0%	OM 83%	STI 0%	Charcot 17%
	MRI			
	Negative 20%	OM 65%	STI 5%	Charcot 10%

**Table 7:** Different therapeutic strategies in patients with final imaging diagnosis of STI.

Therapeutic Strategy in STI	Diagnosis according to Imaging Modalities (%)			
No treatment 1.7%	WBC			
	Negative 0%	OM 100%	STI 0%	Charcot 0%
	FDG (no patients)			
	MRI (no patients)			
Conventional wound care and topic antibiotic treatment 15%	WBC			
	Negative 78%	OM 0%	STI 22%	Charcot 0%
	FDG (no patients)			
	MRI (no patients)			
Systemic antibiotic treatment 68.3%	WBC			
	Negative 19%	OM 23%	STI 39%	Charcot 19%
	FDG (no patients)			
	MRI			
	Negative 15%	OM 15%	STI 47%	Charcot 23%
Surgery (debridement or amputation) 15%	WBC			
	Negative 14%	OM 0%	STI 72%	Charcot 14%
	FDG (no patients)			
	MRI			
	Negative 0%	OM 33%	STI 66%	Charcot 0%
